# An overview of traditional Chinese medicine affecting gut microbiota in obesity

**DOI:** 10.3389/fendo.2023.1149751

**Published:** 2023-03-01

**Authors:** Donghui Li, Weiwei Tang, Yanyan Wang, Qi Gao, Hongwei Zhang, Yu Zhang, Yuliang Wang, Yongyi Yang, Yingming Zhou, Yike Zhang, Haonan Li, Shuo Li, Hong Zhao

**Affiliations:** ^1^ College of Pharmacy, Jiamusi University, Jiamusi, China; ^2^ Department of Emergency Surgery, The First Affiliated Hospital of Jiamusi University, Jiamusi, China

**Keywords:** traditional Chinese medicine, gut microbiota, obesity, short-chain fatty acids (SCFAs), active ingredients

## Abstract

Obesity, a chronic metabolic disease with a complex pathophysiology, is caused by several variables. High-fat diets lead to the disruption of the gut microbiota and impaired gut barrier function in obese people. The dysbiosis and its metabolites through the intestinal barrier lead to an imbalance in energy metabolism and inflammatory response, which eventually contributes to the development of chronic diseases such as diabetes, hypertension, and cardiovascular disease. Current medicines are therapeutic to obesity in the short term; however, they may bring significant physical and emotional problems to patients as major side effects. Therefore, it is urgent to explore new therapeutic methods that have definite efficacy, can be taken for a long time, and have mild adverse effects. Numerous studies have demonstrated that traditional Chinese medicine (TCM) can control the gut microbiota in a multi-targeted and comprehensive manner, thereby restoring flora homeostasis, repairing damaged intestinal mucosal barriers, and eventually curbing the development of obesity. The active ingredients and compounds of TCM can restore the normal physiological function of the intestinal mucosal barrier by regulating gut microbiota to regulate energy metabolism, inhibit fat accumulation, affect food appetite, and reduce intestinal mucosal inflammatory response, thereby effectively promoting weight loss and providing new strategies for obesity prevention and treatment. Although there are some studies on the regulation of gut microbiota by TCM to prevent and treat obesity, all of them have the disadvantage of being systematic and comprehensive. Therefore, this work comprehensively describes the molecular mechanism of obesity mediated by gut microbiota based on the research state of obesity, gut microbiota, and TCM. A comprehensive and systematic summary of TCM targeting the regulation of gut microbiota for the treatment of obesity should be conducted in order to provide new strategies and ideas for the treatment of obesity.

## 1 Introduction

Obesity is a chronic metabolic disease that is caused by excessive accumulation and abnormal distribution of body fat due to the imbalance of energy intake and consumption (1). Research shows that obesity has become a worldwide public health problem. More than 2 billion adults are overweight or obese worldwide, and the prevalence of obesity continues to rise globally (2, 3). Obesity will increase the risk of heart disease, diabetes, digestive disorders, and several types of cancer (4). At present, there are two main types of drugs that have been clinically proven to help with weight loss. One type is the central nervous system drugs, such as cloxacillin, but these drugs have side effects including insomnia, constipation, and anxiety (5). Another type of drug works on the non-central nervous system, such as orlistat, which has the same side effects (insomnia, constipation, and anxiety) (6). According to modern medicine, the pathogenesis of obesity is complex and involves dietary habits, genetic factors, and environmental factors (7). An increasing number of studies have shown that gut microbiota, as a key environmental factor, could contribute to the occurrence and development of obesity (8).

**Figure 1:**
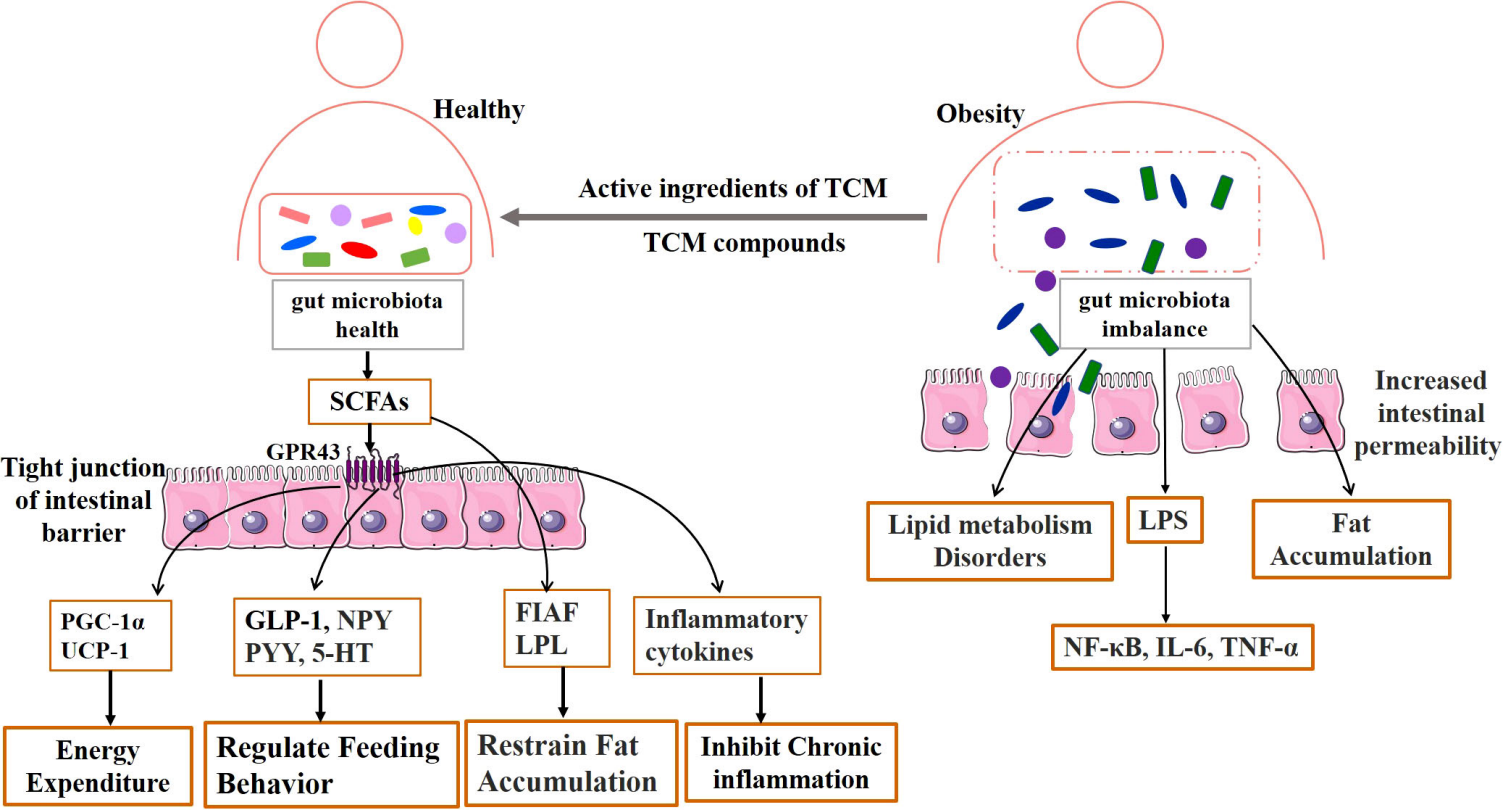
Mechanism diagram of TCM affecting gut microbiota in obesity.

The gut microbiota refers to the microorganisms inhabiting the human gastrointestinal tract. The gut microbiota consists of many interacting symbiotic bacteria, which is considered to be an endocrine organ involved in maintaining energy homeostasis and host immunity. The bioactive metabolites of the gut microbiota can affect the physiological effects of the host (9–12). In humans, the gut microbiota is a complex and dynamic ecosystem that represents approximately 1 kg of our body weight (13). Under normal conditions, the gut microbiota works in harmony with the host and participates in the regulation of many physiological functions of the host, such as nutrient and substance metabolism, food digestion and absorption, the formation of a biological barrier in the intestinal mucosal epithelium, and boosting host immune function (14, 15). Furthermore, healthy gut microbiota produces short-chain fatty acids (SCFAs) to repair and promote intestinal function, and to effectively inhibit the growth of spoiled bacteria in the gut and improve the intestinal environment (16). However, dietary habits or environmental factors affect the composition of gut microbiota, and long-term consumption of improper diet may affect metabolism (17). Several studies have found that antibiotics can change the intestinal mucosa bacterial composition, reduce colonization resistance, and damage the intestinal mucosal barrier by reducing the abundance of gut microbiota (18–20). The high-fat and high-protein diet altered the community structure of lactase bacteria in the intestinal mucosa and decreased the abundance of the critical lactase bacteria (21). The dysbiosis of the gut microbiota may be involved in the pathogenesis of obesity through multiple mechanisms, including disruption of energy homeostasis, lipid synthesis, and storage, central regulation of appetite and feeding behavior, and chronic low-grade inflammation (13, 22, 23). Therefore, regulation of gut microbiota and improvement of intestinal dysbiosis are regarded as key directions for the treatment of obesity, as shown in **Figure 1**.

Traditional Chinese medicine (TCM) has thousands of years of clinical experience in the treatment of obesity, and its benefits are safe, mild, and long-lasting. The majority of TCM is taken orally, and the active ingredients in the TCM interact with the gut microbiota as they enter the gastrointestinal tract. The growth and proliferation of certain microbiota can be stimulated or inhibited by TCM. For example, the Wu et al. study found that *Lilium lancifolium* can promote the growth of *Lactobacillus* spp. and *Bifidobacteria* spp., and inhibit the growth of total bacteria in the intestines of normal mice (24). Qiweibaizhu powder could overcome the influence of dysbacteriosis and could lead to the recovery of intestinal mucosal microbiota homeostasis (25). Folium senna decoction gavage increased intestinal microbiota diversity; *Bacteroides vulgatus*, *Helicobacter ganmani*, *Lactobacillus murinus*, *Microbacterium dextranolyticum*, and *Klebsiella pneumoniae* were significantly enriched, while *Candidatus arthromitus* sp. and *Lactobacillus johnsonii* were significantly depleted (26, 27). Qiweibaizhu powder has a positive effect on the recovery of bacterial lactase gene diversity to normal levels and increases the abundance of *Lysobacter* and *Eukaryota* (28). The Qiwei Baizhu Powder decoction and QWBZP-TG promoted the proliferation of *Lactobacillus* and inhibited the growth of *Proteus*, *Clostridium*, *Eubacterium*, *Facklamia*, and *Escherichia* (29). The active ingredients in the Gegenqinlian decoction may increase the microbial activity of the intestinal mucosa of mice and reduce the microbial activity of the intestinal contents through multiple targets (30). This review will focus on recent advances in the connection between gut microbiota and obesity, and the role of gut microbiota in the treatment of obesity is clarified, in order to find new ideas and research directions for the treatment of obesity and related diseases.

## 2 Mechanisms of obesity mediation by gut microbiota

The gut microbiota colonizes the intestines and has important physiological functions. Studies have shown that obesity is associated with a decrease in gut microbiota diversity, gene expression of gut microbiota, and alteration of metabolic pathways (31, 32). Compared with healthy people, the gut microbiota of obese people usually shows changes at the phylum, family, and genus levels (33, 34). The imbalance of gut microbiota may lead to obesity, which may be related to energy balance, regulation of feeding behavior, fat storage, and inflammation. The mechanisms of gut microbiota-mediated obesity are discussed in this review.

### 2.1 Characteristics of obese gut microbiota

The first evidence for a link between gut microbiota and obesity was from studies of germ-free mice. In 1983, Wostmann et al. first discovered that germ-free rats needed more energy to maintain their body weight compared to wild rats, but the precise mechanism was unknown at the time (35). In 2004, Gordon et al. observed that gut microbiota can affect energy absorption from the diet and energy storage in mice, and found that the proportion of *Firmicutes* was higher and the proportion of *Bacteroidetes* was lower in the guts of obese people (36). Numerous studies have demonstrated that the structure and abundance of gut microbiota are altered in obese people compared to healthy people. Obese people show lower gut microbiota diversity than healthy people and show significant overall obesity and dyslipidemia (37–39).

Numerous studies have demonstrated that the meat-based Western diet has a negative impact on the richness and function of gut microbiota. Being high in animal-derived protein and low in vegetables and fruits, the Western-style diet leads to a significant decrease in the numbers of total bacteria and commensal *Bifidobacterium* and *Eubacterium* species (40). A diet rich in fat, with high consumption of red meat and refined carbohydrates, may have a direct effect on the immune system causing a structural and functional modification of the gut microbiota (41). Lard and vegetable blend oil diet affected the composition of the intestinal microorganisms and the functions of digestive enzymes (42). It was found that more *Firmicutes* than *Bacteroidetes* caused the body to absorb more calories from food and more easily convert calories into fat to accumulate under the skin (43, 44). The gut of obese people is rich in archaea, which can oxidize hydrogen produced by *Prevotella*, thus accelerating the fermentation of polysaccharides and causing the body to absorb more energy (45). According to Petersen et al. (46), *Clostridium* was reduced in obese people and some of the *Clostridium* were able to absorb fat and reduce obesity in mice. The levels of *Ackermanella* are significantly decreased in obese people, which reduces metabolic disturbances caused by high-fat diets, improves fat accumulation, and reduces inflammatory responses (47). In summary, the gut microbiota changes in obese people are mainly shown as the increase in bacteria that cause inflammation and fat production and the decrease in bacteria that inhibit obesity and inflammation. Therefore, it can play a role in the treatment of obesity by regulating the diversity and abundance of the dominant gut microbiota, inhibiting adipogenesis, and the inflammatory response.

### 2.2 Regulation of energy absorption

**Figure 2:**
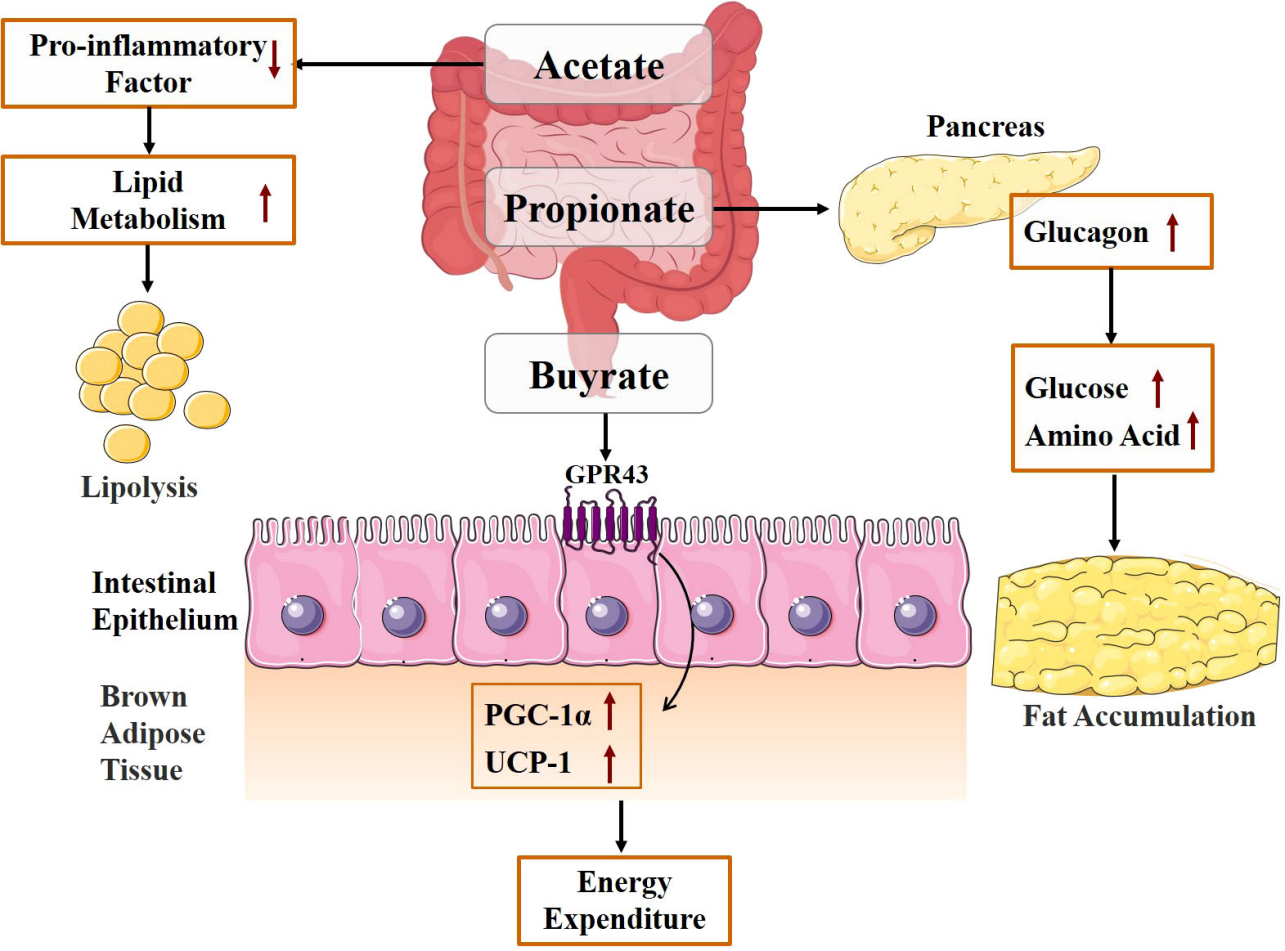
Mechanism diagram of energy absorption mediated by gut microbiota metabolites.

Gut microbiota participates in energy metabolism and absorption under normal and pathological conditions. The obesity-associated gut microbiome can increase the capacity for energy harvest from the diet, thus contributing to increased adipose tissue storage of the host, especially the white adipose tissue (WAT) (48). SCFAs, mainly acetate, propionate, and butyrate, are produced by the gut microbiota through the metabolism of complex dietary plant polysaccharides (49, 50). As one of the primary metabolites of the intestinal microbiota, SCFAs can lower intestinal pH to a certain extent, thereby inhibiting the viability of pathogenic microbiota from promoting intestinal microecological balance and maintaining the integrity of the intestinal mucosal barrier (51). SCFAs are an important source of energy for the host, contributing up to 70% of the daily energy supply for herbivores and 10% of the total daily energy needed for omnivores (13). Among them, butyric acid is a significant source of energy for the epithelial cells of the human colon and cecum (52). As depicted in **Figure 2**, SCFAs not only provide direct energy but also raise energy expenditure and encourage lipolysis. Acetate can have a beneficial effect on host energy metabolism by reducing levels of pro-inflammatory cytokines, increasing energy expenditure and lipid oxidation, and promoting lipolysis (53). Butyrate promotes fatty acid oxidation and increases energy consumption by acting on G protein-coupled receptor 43 (GPR43) and upregulating the expression of peroxisome proliferator-activated receptor coactivator-1 alpha (PGC-1α) and uncoupling protein 1 (UCP-1) expression in brown adipose tissue (BAT) (54). SCFAs are typically considered to have beneficial effects on the body. However, some research suggests that excessive SCFAs may have a negative impact on the body. Clinical studies by Tirosh et al. (55) showed that consuming a propionate-containing diet causes the levels of glucagon in the blood, which raises the risk of obesity. Therefore, gut microbiota metabolites can have both positive and negative effects on the host. If we want to regulate the energy absorption of the body by regulating the metabolites of the intestinal flora, the concentration should be taken into consideration.

### 2.3 Regulation of feeding behavior

**Figure 3:**
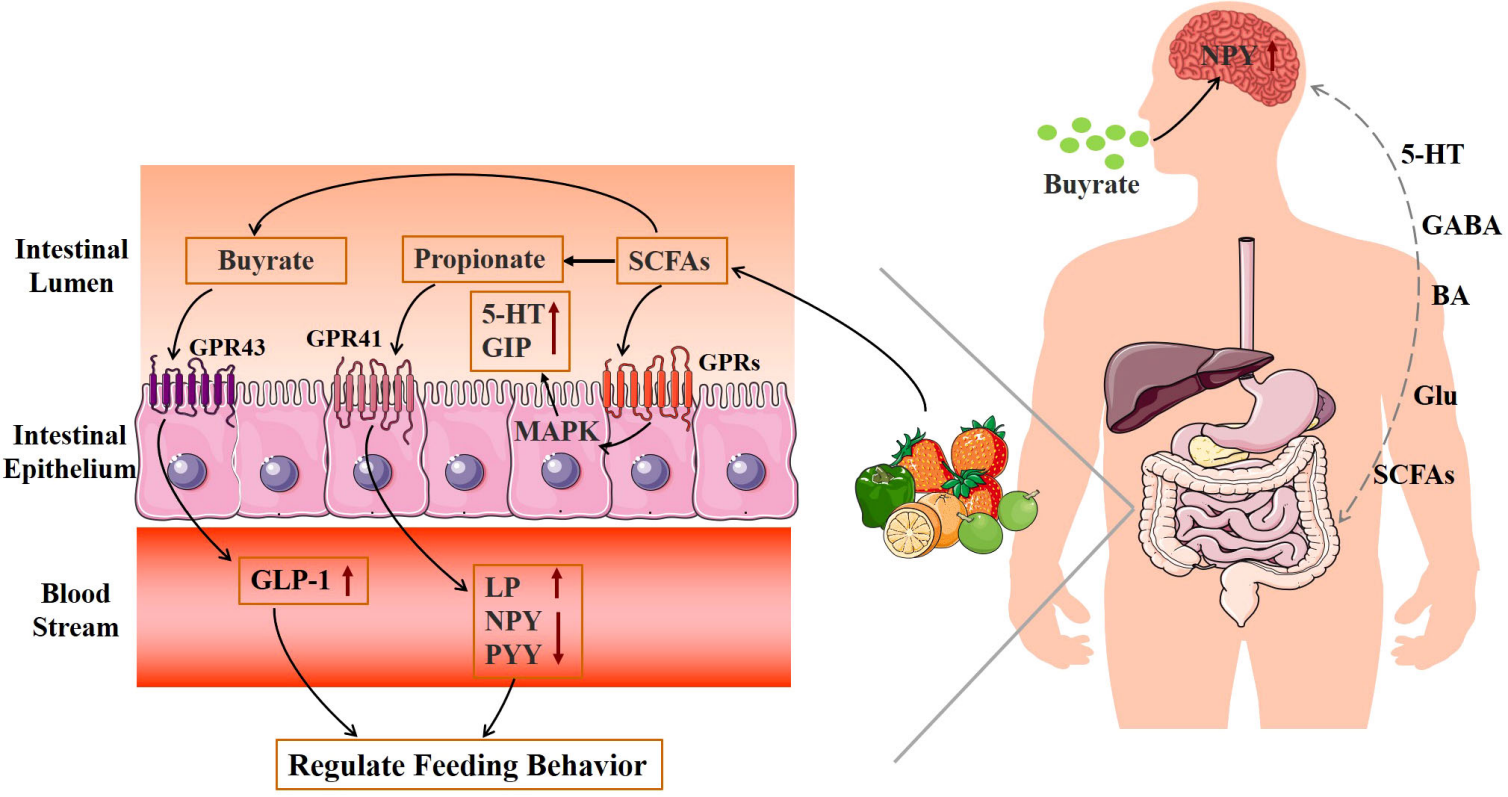
Mechanism diagram of gut microbiota metabolites regulating feeding behavior.

The brain is the main part of the appetite regulation neural pathway, which directly regulates feeding behavior. Gut microbiota produces specific metabolic compounds, which interact with the central nervous system, such as glutamic acid, bile acids, 5-hydroxytryptamine (5-HT), SCFAs, and GABA (Glu) (**Figure 3**) (56, 57). These neuroendocrine components and their receptors have a role in controlling central appetite and feeding behavior by mediating the bidirectional communication between the brain and the digestive system (58). Through the “brain–gut–bacteria” axis, SCFAs can control the body’s energy metabolism to control the GPRs pathway, activate the MAPK signal pathway in intestinal epithelial cells, promote the secretion of 5-HT and gastrointestinal peptides (GIPs) in the intestine, increase satiety, decrease gastric emptying and intestinal movement, affect energy intake, and decrease body weight (53). Propionate in SCFAs activates GPR41, increases leptin (LP) levels, reduces the content of neuropeptide Y (NPY), which has the effect of enhancing appetite, and inhibits the secretion of peptide YY (PYY), which has the effect of appetite (59, 60). Butyrate reduces obesity by acting on GPR43 to increase plasma GLP-1 levels, controlling appetite through the central nervous system, and reducing food intake (60, 61). In addition, oral butyric acid also inhibits the hypothalamic expression of NPY and pro-appetitive neural activity, and decreases food intake (62). According to Frost et al. (63), acetate can reduce hunger by increasing the amount of GABA in mice’s brains. Therefore, the production of SCFAs can be controlled by regulating the structure and activity of the intestinal flora to achieve obesity treatment.

### 2.4 Regulation of fat accumulation

**Figure 4:**
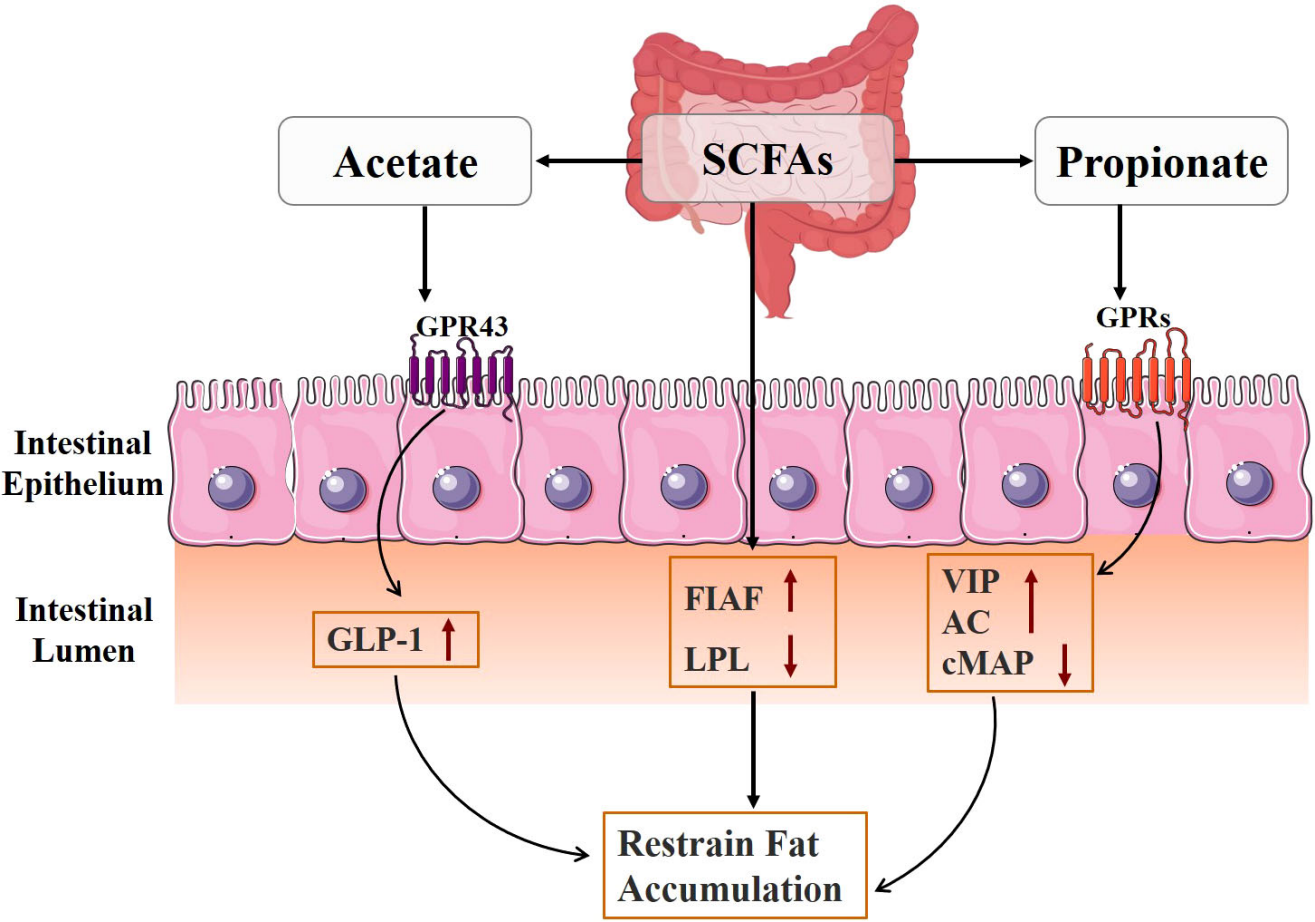
Mechanism diagram of gut microbiota metabolites regulating fat accumulation.

The regulation of genes related to fat accumulation is greatly influenced by intestinal flora and its metabolites (**Figure 4**) (64). Fasting-induced adipokines (FIAFs) inhibit adipose tissue storage by inhibiting lipoprotein lipase (LPL) activity, reducing extracellular triglyceride hydrolysis and fatty acid uptake (65, 66). Studies have shown that SCFAs can inhibit fat accumulation in the body, which may be related to their ability to activate the expression of FIAF and inhibit the expression of LPL, thus preventing the triglyceride cycle (67). In addition, acetate also prevents fat accumulation by stimulating GLP-1 production and activating GPR43 in the gut (68). Propionate regulates glucose and lipid metabolism by binding to and activating neuronal GPRs, causing the release of vasoactive intestinal peptide (VIP) from the submucosal plexus, activating adenylate cyclase (AC), and reducing cAMP levels (69).

### 2.5 The chronic mild inflammatory response

At present, obesity and obesity-related diseases are recognized as systemic chronic low inflammation by the medical community. Recent research has demonstrated that Gram-negative bacteria in the gut microbiota of obese people can create lipopolysaccharides (LPS) and bind to toll-like receptor 4 (TLR-4), activating nearby and distant pro-inflammatory cascades and releasing inflammatory factors (70, 71). TLR4 is an important binding regulator of LPS, which is overexpressed in intestinal mucosal epithelial cells after stimulation of activation, inducing the production of various pro-inflammatory factors and causing and maintaining obesity-type low-level inflammation in the body (72). In addition, damage to the intestinal mucosal barrier can also lead to inflammation. The changes in intestinal flora increase intestinal permeability and promote LPS to enter the blood to activate the NF-κB signal pathway, which leads to chronic inflammation, endotoxemia, and increased fat accumulation, and ultimately leads to metabolic syndrome and obesity (73). The high-fat diet could induce the interactions between *Thermoactinomyces*, *Staphylococcus*, and intestinal inflammation (74).

**Figure 5:**
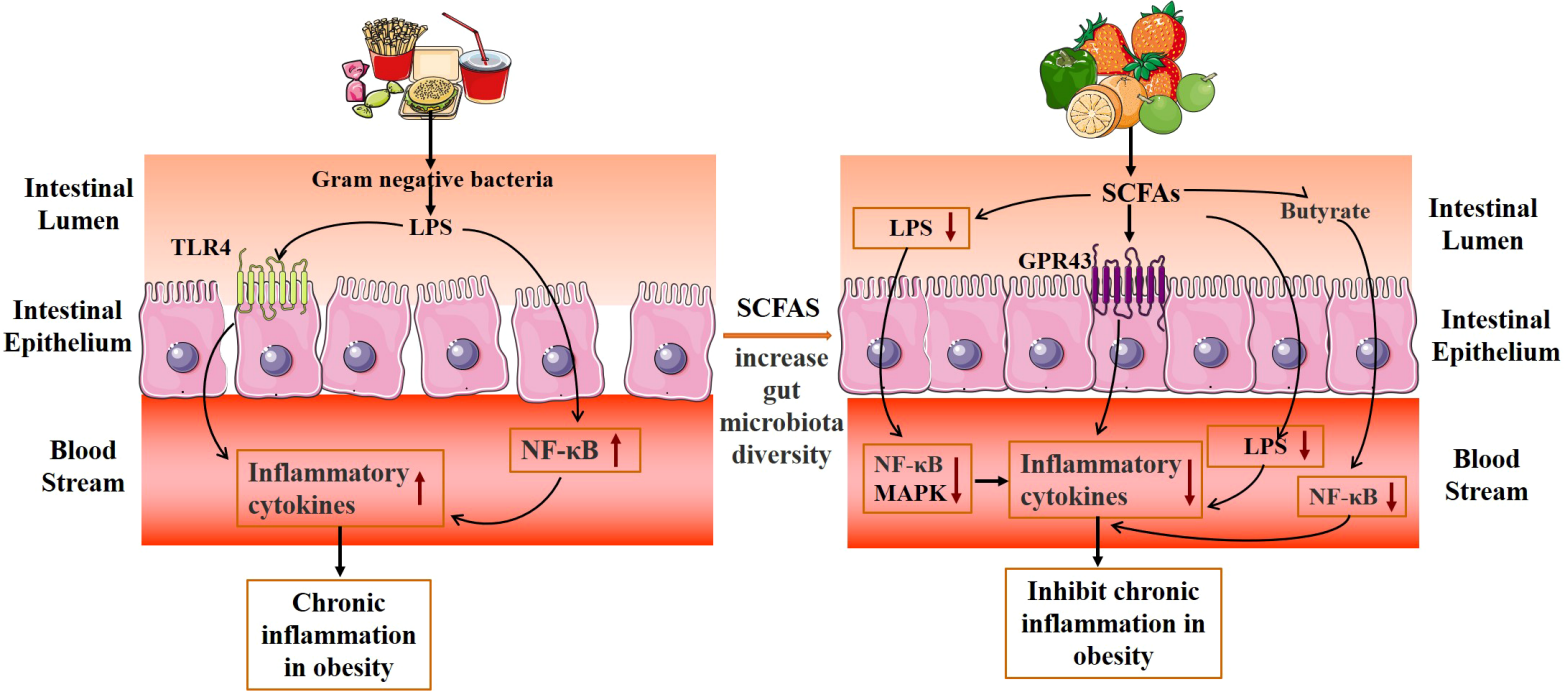
Mechanism diagram of gut microbiota metabolites inhibiting the chronic mild inflammatory response.

Gut microbiota and its metabolites mediate the chronic inflammatory response and inhibit obesity (**Figure 5**). The main mechanisms are as follows: (1) Promote the proliferation of intestinal epithelial cells, reduce the apoptosis of intestinal epithelial cells, and maintain the intestinal mucosal barrier. (2) Regulation of inflammation-related gene expression and reduction of inflammatory response. SCFAs are important signaling molecules to regulate intestinal mucosal immunity, inhibiting LPS or TNF-α-induced inflammatory responses, in which the mechanism is related to the regulation of NF-κB and MAPK signaling pathways (75). The study also discovered that additional SCFA supplementation could enhance mitochondrial activity in brown fat cells, inhibit chronic inflammation, increase the expression of G protein-coupled receptors (GPR43 and GPR41), promote the oxidation of free fatty acids and the hydrolysis of triglycerides, and decrease the body weight of obese mice (76). SCFAs inhibit the transport of toxic substances by maintaining the stability of the intestinal barrier, reducing the concentration of LPS in the blood, and reducing the inflammatory response (77). Acetate improves epithelial cell-mediated intestinal defense and enhances intestinal integrity, thereby protecting the host from lethal infections (78). Butyrate inhibits the activity of macrophages, dendritic cells, neutrophils, and T cells, which lessens inflammation of the intestinal tissue (79, 80). In addition, butyric acid can also block the activity of the transcription factor NF-B and decrease the expression of inflammatory factors like IL-6 and IL-12, which inhibits the inflammatory response in the intestine (81).

In conclusion, the development of obesity is closely correlated with gut microbiota. Human obesity and several numbers of other disorders are brought about by gut microbial dysbiosis. Healthy intestinal flora and its metabolites can play a potential role in preventing or treating obesity by regulating energy absorption, feeding behavior, fat accumulation, and inflammatory reaction.

## 3 The mechanism of TCM affecting gut microbiota to inhibit obesity

The role of regulation of gut microbiota by TCM in the treatment of obesity and related diseases has been confirmed. Several studies have found that the TCM compound, single Chinese medicine, and its active ingredients act on gut microbiota and its metabolites, and play a role in controlling energy metabolism absorption, feeding behavior, and fat storage, and in assuaging chronic delicate inflammation; therefore, it can be used in the treatment of obesity and other related diseases, as shown in **Table 1**.

**Table 1:** Study on the intervention mechanism of TCM affecting gut microbiota in obesity.

Mechanism	Chinese medicine and formula	Target/medium	Reference
The expression of genes that control energy absorption such as GPR43, TC, and TG	Ginsenoside	SCFAs, GPR41, GPR43, GPR109A	(82)
	Root of *Atractylodes macrocephala* Koidzumi	PGC-1α, UCP1	(83)
	Extract of propolis	IL-1β, IL-6, IL-10	(84)
	Polygala tenuifolia extract	SREBP1C, TG, SAA1	(85)
	*Cordyceps guangdongensis*	SCFAs, TC, TG, ALT	(86)
	Lingguizhugan formula	TC, TG, FFA	(87–89)
	Erchen decoction	IRS1, AKT, PKA, HSL	(90)
	Xiexin tang	SCFAs, ACK, BUT, PGC-1α, AMPK	(91)
	Shenlian decoction	/	(92)
The levels of PYY and GLP-1 increase, improve diversity of gut microbiota and the intestinal barrier function, and decrease food intake	Berberine Curcumin	Pla2g2a, PYY, Muc2	(93)
	Grape seed proanthocyanidins	GLP-1	(94)
	Ginsenoside	PYY, NPY, Y2 receptor	(95)
Increases the level of SCFA increase, regulates the abundance of gut microbiota, improves disorders of lipid metabolism, and inhibits fat accumulation	Ganoderma amboinense polysaccharide	SCFAs, TC, TG	(96)
	Rutin	IWAT, UCP1, SCFAs, BUT	(97)
	Heat-treated adzuki	SCFAs	(98)
Inhibits pathways leading to the production of pro-inflammatory cytokines; reduces LPS, thereby reducing chronic low-grade inflammation	Rhizoma Atractylodis Macrocephalae	HDL, IL-6, TNF-α	(99)
	Berberine	CD14, IL-1, IL-6, TNF-α	(100)
	Polysaccharide from the sclerotium of *Poria cocos*	PPAR-γ	(101)
	Polysaccharide from sporoderm-broken spore of *Ganoderma lucidum via* gut microbiota regulation	SCFAs, GPR43, GPR41, IL-1β	(102)
	Polysaccharide from Sijunzi decoction	SCFAs	(103)
	Effects of shenling baizhu	SCFAs, TLR4, IL-1, TNF-α	(104)
	JinQi Jiangtang	SCFAs, MCP-1, IL-6, TNF-α	(105)

### 3.1 Study on the mechanism of active ingredients of TCM affecting gut microbiota to inhibit obesity

#### 3.1.1 Regulation of energy metabolism and absorption

The anti-obesity effect of TCM and its active ingredients is closely related to the regulation of gut microbiota. The active ingredients of TCM can affect the absorption of energy metabolism in the body by improving the abundance of gut microbiota and the expression of related signal pathway proteins. Zhang et al. (82) found that ginsenosides, the active ingredient of ginseng, improved the structural composition of the gut microbiota, increased the concentration of SCFAs and receptor proteins (GPR41, GPR43, and GPR109A), and enhanced the gut barrier completeness. Song et al. (83) found that the root of *Atractylodes macrocephala* Koidzumi extract can prevent diet-induced obesity and glucose intolerance in mice. Its mechanisms are related to increasing the number of brown fat cells, increasing PGC-1 α and UCP1 expression, and promoting the energy metabolism of brown fat tissue. Propolis ethanol extract reduced high-fat diet-induced obesity by regulating the abundance of gut microbiota and improving glucose tolerance and lipid distribution in high-fat diet rats (84). According to Wang et al. (85), *Polygala tenuifolia* extract inhibits high-fat diet-induced lipid accumulation in obese mice by modulating the composition of gut microbiota and regulating the levels of transcription factors of lipid oxidation. Li et al. (106) found that *Dendrobium officinale* can improve the diversity of intestinal mucosal flora and influence the gut microbiota to positively affect high-fat diet-induced negative effects in mice. Asparagus can affect the diversity of bacteria in the intestinal mucosa of mice fed a high-fat diet, and achieve a lipid-lowering effect by regulating the intestinal microecology of mice fed a high-fat diet (107). Baohe pills can restore the number of intestinal flora to a certain extent and improve the activities of various digestive enzymes including protease and amylase in mice fed a high-fat and high-protein diet (108).

#### 3.1.2 Regulation of feeding behavior

The active components of TCM can improve the abundance of gut microbiota and the expression of associated signaling pathway protein in obese mice to regulate feeding behavior and thus play a role in treating obesity. The active components of TCM can alter feeding behavior, increase microbiota abundance, and consequently increase the production of proteins involved in linked signaling pathways in obese mice. According to Neyrinck et al. (93), berberine and curcumin can improve intestinal barrier function, increase the number of intestinal probiotics, upregulate the expression of the innate immunity genes Pla2g2a and PYY, and decrease food intake in obese mice. Casanova-Martí et al. (94) discovered that grape seed proanthocyanidins can suppress appetite and promote weight reduction by controlling the quantity of gut microbiota in rats, raising serum GLP-1 levels, and upregulating GLP-1 mRNA and protein expression in ileum and colonic tissues. According to Lin et al. (95), diet-induced obese mice’s appetites were suppressed and their weight was decreased because Rb1, the active component of ginseng, was able to upregulate the expression of PYY mRNA in the intestine and downregulate the expression of NPY in the gut.

#### 3.1.3 Regulation of fat accumulation

The active components of TCM effectively reduce fat formation by high-fat diets by controlling the homeostasis of gut microbiota and microbial metabolites. In mice given a high-fat diet, Ren et al. (96) discovered that *Ganoderma lucidum* polysaccharides efficiently lower blood lipid concentrations, regulate the abundance of gut bacteria and various critical pathways, and successfully relieve fat formation. According to Cheng et al. (97), Rubin may greatly raise the amounts of SCFAs and SCFA-producing enzymes in the feces of obese mice, prevent fat accumulation, treat glucolipid metabolic problems, and cause obese animals to lose weight. Heat-treated adzuki bean protein hydrolysates can assist to prevent and treat obesity and its associated consequences, as well as increase the diversity of gut bacteria in mice fed a high-fat diet (98).

#### 3.1.4 Alleviation of chronic mild inflammatory response

TCM and its active components can significantly improve the intestinal environment, enhance intestinal barrier function, and control the production of inflammatory markers associated with obesity during intestinal metabolism. Wang et al. (99) showed that fermented Rhizoma Atractylodis Macrocephalae can regulate the gut microbiota, improve the growth of probiotics, alleviate the inflammatory response, and restore the intestinal epithelial barrier function and exert weight loss effects. According to Cao et al. (100), berberine may help people lose weight by controlling the abundance of their gut bacteria and lowering levels of pro-inflammatory cytokines like IL-1, IL-6, and TNF-α. Insoluble polysaccharides from *Poria cocos* sclerotium can significantly reduce the weight of obese mice by increasing the abundance of intestinal flora, improving the integrity of intestinal mucosa, and activating the intestinal PPAR-γ pathway to reduce inflammatory response (101). *G. lucidum* polysaccharides improve dysbiosis of the gut microbiota and maintain intestinal barrier function to inhibit TLR4/Myd88/NF-κB signaling pathway expression in adipose tissue, thereby exerting treatment of obesity (102).

### 3.2 Mechanism of TCM compounds affecting gut microbiota to control obesity

#### 3.2.1 Regulation of gut microbiota abundance and lipid metabolism

The TCM compounds can regulate the abundance of gut microbiota and lipid metabolism to treat obesity. In the mechanism involving the regulation of gut microbiota abundance, SCFAs, and genes involved in lipid metabolism, the Cordyceps guangdongensis lipid-lowering formula dramatically reduced weight and fat accumulation in high-fat diet mice (86). By lowering total cholesterol (TC) and triglyceride (TG) levels and increasing the quantity and variety of gut microbiota, the Jian Pi Tiao Gan Yin medicine helps mice with obesity (109). By controlling the distribution and relative abundance of gut microbiota, enhancing the small intestinal villi, and altering the metabolic pathways linked to obesity, the Lingguizhugan decoction (consisting of Poria, Ramulus Cinnamomi, Rhizoma Atractylodis Macrocephalae, and Radix Glycyrrhizae) aids in weight loss (87–89). By changing the composition of the gut microbiota and the relative abundance of bacteria in obese rats, the Erchen decoction (consisting of Radix et Rhizoma Panax, Radix tangerine, Poria, and Radix Glycyrrhizae) can improve lipid metabolism disorders and change the function of the gut microbiota and cause weight loss (90). In addition to improving obesity, Xiexin Tang (a combination of rhubarb, Huang Lian, and Scutellaria) increases the activity of crucial enzymes involved in the synthesis of SCFAs, enhances the gut microbiota’s capacity to metabolize SCFAs, and decreases energy intake while increasing energy expenditure (91). The Shenlian (SL) decoction (composed of Coptis and ginseng) is considered a potential drug for weight loss, which can decrease the expression of flora associated with LPS biosynthesis, increase the diversity and abundance of gut microbiota, and regulate metabolic disorders (92). Daesiho-Tang is an effective herbal formulation in attenuation of obesity in HFD-fed mice through the alteration of gene expressions and modulation of intestinal microbiota (110). The TCM formula (CoTOL) (composed of Glabrous Greenbrier Rhizome, Dioscorea septemloba Thunb, Curcuma Longa, and so on) has beneficial effects on hyperuricemia and overweight, which may be attributed to regulating material metabolism and improving the structure or function of gut microbiota (111). The TCM Formula Kang Shuai Lao Pian (composed of glutinosa, ginseng, japonicas, and so on) could improve HFD-induced obesity, glucose tolerance disorder, and gut dysbiosis (112).

#### 3.2.2 Regulation of gut microbiota abundance and reduction of chronic inflammation

The Sijunzi decoction, which contains ginseng, *Atractylodes macrocephala*, Poria, and Glycyrrhiza glabra, can have immunomodulatory effects by regulating the number of gut microbiota and SCFA levels (103). The shenling baizhu powder herbal formula (composed of lotus seed meat, coix kernel, sand kernel, bellflower, poria, ginseng, and licorice) reduced weight and serum total cholesterol levels and repaired intestinal mucosa in high-fat diet rats, and the mechanism may be related to reducing TLR4-related protein expression, decreasing the levels of inflammatory factors such as TNF-α and IL-1, and increasing the relative abundance of gut microbiota (104). Jinqi Hypoglycemic Tablets (composed of Huang Lian, Huang Qi, and Jin Yin Hua) regulate the gut microbiota and promote the production of SCFAs in HFD mice by a mechanism related to enhancing intestinal barrier function and reducing host inflammatory response (105). The Shen-Yan-Fang-Shuai formula (composed of Astragali Radix, Radix Angelicae Sinensis, Rheum Officinale Baill, and four other herbs) treatment prevented weight gain, low-grade inflammation, and insulin resistance in HFD mice (113). The Gegenqinlian decoction could intervene from inflammatory response through multiple targets and multiple channels to adjust the balance of intestinal mucosa flora (114).

## 4 Conclusion

Gut microbiota is closely related to the occurrence and development of obesity and related metabolic diseases. TCM can improve the metabolic disorder of obesity by regulating the imbalance of gut microbiota. Gut microbiota can be used as a new target for the prevention and treatment of obesity and to improve obesity and its metabolic diseases by correcting gut microbiota disorders. This study primarily summarizes how TCM treats obesity by regulating the flora in the intestines. The active ingredients of TCM and its compound can lower blood lipid levels and fat accumulation in patients by controlling the distribution of gut microbiota, enhancing the capacity of gut microbiota metabolism to generate SCFAs, reducing inflammatory damage, and improving the energy metabolism of the body, thereby improving obesity. Further study of the relationship between TCM and gut microbiota will help to enrich the theory of TCM and develop TCM preparations that target the gut microbiota. However, the research on the interaction between TCM and gut microbiota is complex and still in the initial stage. Many key scientific issues need to be further explored. The internal environment of gut microbiota is complex, and different types of bacteria produce different metabolic enzymes, which makes it difficult to explain the biotransformation of active ingredients of TCM and gut microbiota. In the future, the molecular mechanism of gut microbiota regulation should be further explored. It is helpful for us to understand that TCM directly acts on gut microbiota or indirectly regulates the structure of gut microbiota and maintains the balance of intestinal micro-ecology, in order to promote the use of herbal medicine in the prevention and treatment of difficult-to-treat diseases such as obesity.

## Author contributions

DL and WT reviewed a large amount of literature, conducted relevant data analysis, and drafted the manuscript. YYW and QG completed the mechanism diagram. YY, HWZ, YMZ and YW analyzed the relevance of the literature and provided the analysis report. YZ supervised the project and revised the manuscript. YKZ, HL and SL analyzed the data and revised the manuscript. HZ reviewed the article and provided revisions, formal analysis, and manuscript drafting. All authors contributed to the article and approved the submitted version.
